# “All Weather Friends”: How China Transformed Zimbabwe’s Tobacco Sector

**DOI:** 10.3390/ijerph17030723

**Published:** 2020-01-22

**Authors:** Jennifer Fang, Lauren De Souza, Julia Smith, Kelley Lee

**Affiliations:** Faculty of Health Sciences, Simon Fraser University, Burnaby BC, V5A 1S6, Canadajhs6@sfu.ca (J.S.);

**Keywords:** tobacco, China, Zimbabwe, development, aid, globalization, agriculture, tobacco control policies, tobacco farming, tobacco supply

## Abstract

Recent research documents the globalization strategy of the Chinese tobacco industry since the early 2000s and risks posed to global health. There are limited analyses to date of how this strategy is playing out in specific countries. This paper analyses the expansion of the China National Tobacco Company (CNTC) in Zimbabwe, the largest producer of tobacco leaf globally, since the early 2000s, through document analysis. It applies a political economy framework—identifying material, ideational and institutional forces—to demonstrate how CNTC capitalized on the unique features of China-Africa development cooperation to pursue its expansion goals, which threaten global public health efforts to reduce tobacco supply. In a context of economic crisis, CNTC offered substantial resources to revive Zimbabwe’s tobacco industry, promoting a shift to contract farming of its preferred leaf. It benefited from perceptions of state friendship, which it fostered through corporate social responsibility initiatives. Through ties with the Chinese embassy and economic actors, CNTC embedded its interests in development institutions. While contributing to improved foreign exchange earnings and some farmers’ livelihoods, CNTC’s expansion has increased the dependence on China as a development partner and tobacco as a crop, benefitting its “go global” strategy, while contributing to public health and environmental challenges locally and globally. The expansion of the Chinese tobacco industry interests in Zimbabwe offers lessons for global tobacco control and efforts to support alternatives to tobacco growing.

## 1. Introduction

China is the largest tobacco market in the world, with one-third of the world’s smokers consuming 40% of global cigarette production [1]. The market is controlled by the China National Tobacco Corporation (CNTC), a state-owned monopoly and the world’s largest tobacco company. Traditionally focused on the domestic market, the anticipated decline of this market over time has led the company to look outward. Beginning in the early 2000s, the Chinese tobacco industry underwent restructuring and rationalization, consolidated its brand portfolio, developed new brands for foreign markets, and expanded overseas leaf procurement operations. In 2007, CNTC officially announced its “go global” strategy. By 2019, CNTC was operating dozens of subsidiaries worldwide with the aim of creating and supplying foreign markets [2]. Evidence of a shift in business strategy by the Chinese tobacco industry, towards the pursuit of foreign suppliers and markets, raises profound implications for global tobacco control.

It appears that China is seeking to join the ranks of the lead transnational tobacco companies, which have consolidated and focused on global markets since the decline of consumption in so-called traditional markets in North America, Europe, and Australia/New Zealand from the 1960s on [3]. Evidence suggests that China’s ambitions to join these ranks would lead to increased competition, especially in emerging markets in Asia, Africa, and the Middle East, resulting in downward pressures on price, product innovation, and intensified marketing and promotion efforts. Increased competition, in turn, will promote increased supply and consumption and, consequently, adverse health and environmental impacts. Tobacco use contributes to over 7 million deaths annually and is a leading contributor to deforestation in supplying states such as Zimbabwe [4]. Thus, there is an urgent need to better understand the global expansion strategy of the Chinese tobacco industry. However, to date, there has been limited analysis of how this strategy is playing out in specific countries.

China is an increasingly important development partner in sub-Saharan Africa. Between 2000 and 2014, African countries received over 1592 Chinese overseas development assistance projects worth over $34.8 billion US [5]. However, China’s approach to development cooperation is controversial. Critics point out China’s support for “pariah states” and the lack of transparency of its foreign aid programs [6]. Trade and investment, especially through state-owned enterprises (SOEs), are often included in foreign development projects, mixing commercial interests with foreign aid, generating criticism of China as a neo-colonial power [7]. On the other hand, China is praised for its emphasis on “win-win”, or co-development, and engagement with African states as equal partners [7]. China’s policy of non-interference, dating back to 1964, respects state sovereignty, and does not attach conditions to foreign aid and loans [6]. Chinese engagement is often welcomed by African leaders who seek to replicate its domestic success in tackling poverty and public health.

One major component of China’s foreign aid programs is agricultural cooperation, which first started with food aid provision in 1959 [8]. This has since expanded to include technical assistance and related infrastructure construction. Chinese engagements in agriculture in sub-Saharan Africa increased by 175%, from $30 million US in 2009 to $82.47 million US in 2012. Chinese-African agricultural cooperation takes different forms, typical of Chinese foreign aid programs, including traditional aid, joint ventures, and investments, and public-private partnerships [9]. The blurring of aid and commercial interests is justified as essential to sustainability. In 2002, the vice-minister for foreign aid at the Ministry of Foreign Trade and Economic Cooperation declared that “China-Africa agricultural cooperation … must be conducted by enterprises and should be market-oriented” in order to ensure long term development [9] (p. 693). This policy was announced at roughly the same time as the central government’s “go global” strategy, aimed at supporting Chinese enterprises to enter foreign markets [2].

China is one of the few agricultural development partners willing to support tobacco farming [10]. Most donor organizations explicitly exclude support for tobacco farming in their programs, recognizing that tobacco use is a leading contributor to ill health and mortality globally. In addition to the negative health impacts of tobacco products, tobacco farming has been associated with child labor [11], deforestation [12], and green tobacco sickness [13]. While past studies have found tobacco farming to have a negative impact on food security, more recent research suggests the situation is highly nuanced [14]. However, they do agree that tobacco farming is often less profitable than comparable crops and highlight the high labor and inputs costs of growing tobacco [15,16]. The World Health Organization (WHO)’s Framework Convention on Tobacco Control (FCTC), established in 2003, commits state parties (including both China and Zimbabwe) to implement both supply and demand side measures to reduce global tobacco use (one of the leading causes of death worldwide). Most UN agencies and donor organizations also have policies to dissociate themselves from the tobacco industry [17]. For example, the World Bank has a corporate mandatory operational policy “that precludes lending, provision of grants, investing in, or guarantee investments or loans for tobacco production, processing, or marketing” [18]. However, the lower- and middle-income countries that produce the bulk of the world’s tobacco leaf argue, along with the transnational tobacco companies that buy it, that the industry is an important contributor to rural livelihoods, government revenue, and employment [19,20,21]. The tensions between perceived economic benefits of tobacco farming and its related health harms raise critical questions about Chinese state-sanctioned support for tobacco as part of agriculture development.

This paper presents a case study of the expansion of CNTC’s supply-side activities in Zimbabwe, one of the largest exporters of tobacco leaf in the world. Worth $4 billion US in 2018, tobacco is the second most valuable export for the southern African country after gold (worth about $9 US million annually) [22]. The history of the tobacco industry in Zimbabwe, and its role in the agriculture sector, is well-documented. Existing studies, however, focus on the economic benefits generated [23] and the importance of the crop to the livelihoods of approximately 145,725 Zimbabwean farmers [24,25,26]. There have been limited analyses of changes in the Zimbabwean tobacco sector over the past decade. Of particular importance has been the increasing role of Chinese interests in the sector and the implications for farmers, in particular, and global public health, more generally. This paper analyzes Chinese support for Zimbabwe’s tobacco industry within the context of China-Zimbabwe development cooperation and assesses implications for global health. It finds that China’s revitalization of Zimbabwe’s tobacco industry has created new relationships of dependency and provides important lessons for global tobacco control’s growing efforts to support sustainable alternatives.

This study builds on previous research on the political economy of transnational tobacco companies, presenting a unique analysis of a state-owned company’s influence in another jurisdiction. As such, it presents a critical case of the overlap between state political priorities and corporate economic goals, interrogating how globalized corporate structural power operates to constrain government action. This political economy lens illuminates the links between economic development and health policy, as well as the various types of influence of non-state actors within policy processes. In conceptualizing influence, we draw on the framework developed by critical international relations theorist Robert Cox, who argues that policy processes are shaped by three types of forces: material capabilities, ideas, and institutions [27] (discussed further below).

## 2. Materials and Methods

In conducting this case study, we acknowledge that access to primary data on Chinese tobacco industry interests in Zimbabwe is highly limited. Information about the activities of the CNTC, an important state-owned monopoly (generating 7–11% of government revenues [1]) of a country ruled by a one-party system, is tightly controlled. Similarly, the economic importance of the tobacco sector in Zimbabwe, a country ruled by a 37-year dictatorship, has meant little transparency of its operations. As such, this case study is based on a variety of document sources, such as industry reports, news media, publications from civil society organizations, and trade data.

We began by conducting a review of scholarly and grey source literature, published between 1999 when the fast track land reforms began and the end of 2019, to understand the historical context of Chinese foreign policy in Zimbabwe and, more specifically, changes in Zimbabwe’s tobacco sector since the early 2000s. The databases Web of Science, Global Health, Medline, and Google Scholar were searched using the terms Zimbabwe AND tobacco; Zimbabwe AND agriculture; Zimbabwe AND China AND tobacco; China AND Zimbabwe AND agriculture.

To gather data on key actors, recent events, and policy decisions concerning Chinese tobacco interests in Zimbabwe, we searched *Tobacco Journal International*, LexisNexis and AllAfrica.com using the same keywords. We also conducted general English-language Google searches for government and non-governmental organization (NGO) reports using the search terms above. We then performed on-line searches of Chinese-language websites for information on Chinese tobacco interests in Zimbabwe, including the CNTC, Tobacco China, Ministry of Commerce, and Baidu. Documents in Chinese were analyzed in their original text, except where direct quotations were used in the results; these were translated by the lead author. Searches were conducted iteratively, with sources used to generate follow up searches using a snowballing technique. We included—based on criteria that the documents include mention of Chinese actors (state or private) in Zimbabwe’s tobacco industry-16 documents from international organizations, NGOs and think tanks; 16 media articles (four of which were in Chinese), and seven sources from state actors such as the Government of Zimbabwe, China’s Ministry of Commerce and the Chinese Embassy in Zimbabwe. We did not search existing collections of internal tobacco industry documents, which currently do not archive CNTC or Zimbabwean tobacco company documents relevant to the period under study, as the majority of industry documents originated prior to 2000 [28]. The first and second authors first organized the above data into a chronology of events leading to China’s engagement in Zimbabwe’s tobacco industry from the early 2000s onwards. The use of multiple sources, notably Chinese and English-language sources independent of either government, to triangulate findings, increased the reliability of the findings.

In developing a framework through which to analyze instances of Chinese engagement in Zimbabwe’s tobacco sector, we began with the types of influence identified by Cox discussed above: material capabilities, ideational influence, and institutional influence. This framework has been applied in health policy analysis, for example, by Harmer and Buse [29] to assess the influence of BRICS (Brazil, Russia, India, China, and South Africa) countries in global health policy. Within agricultural policy analysis, De Bruyn [30] uses this framework to assess the influence of BRICS countries in agricultural development in Malawi. We build off these previous studies to apply Cox’s broad concepts to analyze the influence of China on Zimbabwe’s tobacco sector; we conceptualize material capabilities as influence generated through the provision of goods and services, such as financial assistance, human resources, and technical assistance. Ideational influences include the ability to shape principles of development cooperation and approaches to addressing development challenges. Institutional influence is generated through relationships between actors and over the types of institutions involved, and modes of cooperation. The first and third authors coded data according to these three types of influence, developing subthemes inductively (see Table 1). Results were reviewed by all of the authors through a collaborative process to achieve consensus.

## 3. Results

### 3.1. Material Capabilities: Bilateral Relationship with Zimbabwe

Tobacco farming in Zimbabwe dates from the colonial era (1888–1980), then known as Southern Rhodesia, with flue-cured Virginia leaf commercially produced on large tracks of farmland by 1910 [31]. The cash crop was grown in large quantities and proved so profitable that it was considered “as valuable as gold or diamonds” [32]. Tobacco was primarily grown by white settlers, who were awarded 51% of the country’s land under the 1930 Land Apportionment Act. White farmers formed the Rhodesia Tobacco Association in 1928, which became a “powerful political force” [32]. Over the next fifty years, production flourished in Southern Rhodesia, which became among the world’s largest producers of flue-cured Virginia leaf, exporting 98% of its leaf.

Following independence in 1980, the Zimbabwean government under President Robert Mugabe came under pressure to redistribute large tracks of commercial farmland still largely owned by white farmers [32]. While many whites left after independence, those who remained continued to control key parts of the economy, including the agricultural sector. With whites representing 1% of the population but owning 70% of the arable land, the controversial Fast-Track program began to be implemented in 2001. Under the policy, commercial farmland was forcibly seized by so-called ‘war veterans’ and youth militia and redistributed to black Zimbabweans. Today, almost no arable land is owned by white farmers. Because of the government’s decision not to stop violence used during the land seizures, Western countries began to impose financial and visa sanctions in 2002, which remain to the present day. The hardships during this period of economic instability included a sharp decline in tobacco farming. New smallholder farmers lacked experience, capital and expertise, and Western sanctions limited access to key inputs. Leaf production plummeted to an all-time low. This was disastrous for the already faltering Zimbabwean economy, given the substantial earnings from tobacco growing.

As Western countries imposed sanctions on Zimbabwe on the basis of human rights violations, China was among a small number of countries that increased engagement during this period of land reform. Bilateral relations date from China’s support for the Zimbabwe African National Unit, headed by Robert Mugabe, during the country’s struggle for independence against British colonial rule [33]. Formal diplomatic ties were then established immediately after independence [34]. During the 1980s, this relationship “grew through loans, [and] projects”, alongside Chinese heritage projects and high-level official visits [35]. By the early 2000s, amid growing international isolation, relations with China became strategically vital politically and economically for Zimbabwe [34,35]. In 2003, Mugabe put forth a “Look East” policy, which aimed to cultivate Asian development partners further.

For China, closer relations with Zimbabwe coincided with the country’s renewed interest in the African continent, formalized by the establishment of the Forum on China-Africa Cooperation in 2000. Sustained high rates of economic growth domestically began to be accompanied by increased attention to the advancement of political and economic goals through foreign policy. This included securing access to vital commodities and other natural resources, growing potential markets, and gaining allies in international forums [36]. With this alignment of interests, bilateral trade grew between the two countries, and China soon became the largest foreign investor in Zimbabwe [36].

### 3.2. Material Capabilities: Bilateral Assistance for Tobacco Farming

In 2004, the Government of Zimbabwe invited a high-level Chinese delegation from the tobacco-growing province of Yunnan to visit Zimbabwe and explore investment opportunities. Subsequently, in 2005, the CNTC subsidiary Tian Ze Tobacco Company (TZTC) was established in Harare, based on a memorandum of understanding signed between the governments of Zimbabwe and China [37], “with the sole purpose of helping revive the country’s tobacco output” [38]. As a state-owned enterprise, TZTC has direct access to Chinese loans and concessions, aiding its entry into Zimbabwe and enabling it to operate without concern for initial financial losses [39]. With a starting investment of US $5 million, TZTC’s goal was to engage in contract farming and participate at auction floor sales to procure quality tobacco leaf for export to China. TZTC would also sell its tobacco to other transnational tobacco companies, including British American Tobacco [40]. Managed through CNTC, TZTC’s presence was expected to meet several of CNTC’s operational objectives: (**i**) to improve its global value chain by directly accessing raw materials at the source; (**ii**) to improve efficiency by minimizing reliance on resellers; and (**iii**) to ensure quantity and quality of tobacco leaf [38]. In addition, TZTC was also expected to represent CNTC’s interests in other leaf-growing countries in the Southern African region [38].

Traditionally, tobacco sales were conducted via three government-operated auction floors, where tobacco farmers brought their leaf and were free to sell to the highest bidding tobacco merchant, who then processed and resold the leaf to tobacco manufacturers [22]. Contract farming was introduced to Zimbabwe in the 2003/2004 growing season, modeled after Brazil. Under this arrangement, a smallholder farmer enters into a contractual agreement with a buying company, such as TZTC, who provides seeds, fertilizer, equipment, and training upfront [22]. These costs are then deducted from the farmer’s earnings at the end of the season. Contract farming has been promoted globally by the tobacco leaf buying companies, including CNTC, as it increases their control over quality and production quantity. The Zimbabwean government supported a shift to this model as a way to address cost-related barriers faced by smallholder farmers wishing to grow tobacco. However, the government also aimed to continue the previous auction floors system as an important avenue to exert control over the foreign exchange in the tobacco industry through pricing [41].

TZTC entered into contractual agreements with 78 farmers during its first season in 2007, procuring 898 tons of tobacco [42]. TZTC collaborated with well-known local tobacco farms to establish the China Tobacco Demonstration Farm in 2008, with hopes of attracting more tobacco farmers [43]. By 2010, it had contracts with 162 farmers producing 9493 tons of tobacco leaf, and by 2012, it was one of the largest contracting firms, working with over 250 farmers [41]. Demand for high-grade flue-cured tobacco from TZTC led to a decline in burley production, which has not been grown in Zimbabwe since the 2013/2014 season [25]. By 2015 TZTC was the single largest exporter to China, buying approximately 40% of Zimbabwe’s tobacco leaf, accounting for 55% of the total export value as TZTC only purchases the highest-grade tobacco leaf [39,44]. It employed nearly 100 regular staff members, with eight Chinese nationals in senior management positions and 87 local employees [41]. It also employed 150 seasonal workers during the harvest season and had contractual agreements with approximately 20,000 farmers annually [38]. Although TZTC’s market share as a tobacco merchant fluctuates year by year, ranging from a low of 9.24% in 2018 to a high of 16.6% in 2015 [25,45], China has remained the main export destination for Zimbabwe’s tobacco [25,45,46,47]. Due to its role in revitalizing the industry and continued prominent market share, TZTC has been described as having “transformed” the Zimbabwean tobacco industry [48].

TZTC initially recruited contract farmers by offering prices higher than that of the auction floors [38], a commonly-used practice to attract and retain farmers [49]. Recent data, however, suggests that prices fluctuate year by year [25,45,46,47]. TZTC mostly works with larger farms, only supporting smallholder farmers through a collaboration with a local contractor, the Mashonaland Tobacco Company [40]. The details of this arrangement are sparse, and it is only known that TZTC funds Mashonaland Tobacco Company to engage smallholder farmers [40]. Media reports of farmers who contract with TZTC list the company’s benefits, such as zero-interest loans, free inputs including fertilizer and pesticides, as well as the provision of equipment, heavy machinery, and technical support [41]. A letter reportedly from a local Zimbabwean farmer published in China’s *People’s Daily* praises TZTC for providing “selfless help” [50]. The farmer is said to be from East Mashonaland who was able to increase his income ten-fold and expand his acreage by more than four times since starting work with TZTC. Initially attracted by the higher prices offered, he also benefits from interest-free loans, free inputs, and much-needed farming equipment such as tractors, power generators, and trucks, as well as technical assistance [43]. However, NGO reports find TZTC contracts include higher interest rates than similar companies, and that TZTC farmers are not consistently provided with safety equipment and training [11]. In recent years, there have been several cases of reported suicides as farmers contracted with TZTC were unable to pay back debts at the end of the growing season [51,52].

### 3.3. Ideas: Cultivating China’s Role as an All-Weather Friend

China’s support throughout Zimbabwe’s transition and crises led the then president Robert Mugabe to praise China as “Zimbabwe’s all-weather friend” [44]. The long-standing relationship between the two states is described as “win-win” and a “friendship built on mutual cooperations” [53] (p. 22). While the benefits of this friendship are not so easily accepted by the Zimbabwean people, and often questioned in the press, they are promoted by both governments [41]. This rhetoric benefits Chinese companies, particularly SOEs like TZTC, by providing legitimacy and authority for their activities in Zimbabwe. The companies, in turn, perpetuate ideas of a mutual beneficial relationship. For example, TZTC is an active participant in Chinese foreign aid projects coordinated through the Ministry of Commerce and has participated in the distribution of donated materials and supported charity clinics organized by Chinese medical teams [38]. In contributing to activities that sustain the idea of friendship between the two states, TZTC strengthens its own reputation and business interests.

TZTC also implements numerous corporate social responsibility (CSR) projects, with focus areas ranging from the environment to supporting education and social causes [38,41]. In 2010, TZTC invested nearly US $1 million to build a school named after Ma Bo, a former employee who had tragically died [54,55]. Built in the major leaf-growing region of Beatrice, the school is called China Tobacco Ma Bo Hope Primary School and is simply known as Dunnolly Primary School in English [44]. The new brick and mortar school building comprising of six classrooms [44] was an improvement from former make-shift classrooms in abandoned poultry sheds or under the trees [55], and significantly improved enrollment and staffing [54]. The Chinese name indicates it is modeled after China’s Project Hope schools, a government initiative targeting rural development [56], with many of its schools sponsored by CNTC branches. Known as “tobacco schools” in China, they are often named after individual cigarette brands or CNTC branches [57]. TZTC is also reported to be supplying the school with over $10,000 US worth of school supplies and sports equipment every year [55]. In 2017, a second investment of $60,000 US was made to build living quarters for teachers [55]. TZTC’s other CSR projects include providing financial support to orphanages, sponsoring academically-gifted children, reforestation projects to address the negative impacts of tobacco curing [38], and emergency relief [58]. A long-standing support of education and children is promoted in company documents stating “[t]he plight of disadvantaged children continues to be Tian Ze Tobacco’s major concern”, and that Tian Ze “view[s] children as the best hope for the future and should, therefore, be afforded equal opportunity to develop their talents” [44]. The support TZTC provides is also celebrated in the tobacco farmer’s letter in China’s *People Daily*, noted above, which credits TZTC with caring for farmers on a personal level through building schools to address the education needs of children brought by hundreds of seasonal workers.

TZTC’s contributions to Zimbabwean society is recognized by both governments, with the Chinese embassy praising and promoting such projects as “selfless”, showing that Chinese businesses are also making positive social impacts to Zimbabwe’s development, and contributing to China-Zimbabwean friendship [54]. The Zimbabwean government has recognized TZTC’s contributions with the “Best Community Development” award distributed by the Ministry of Education in 2013, and the “Best Social Contribution” award by the National Business Bureau [41]. Local media, especially state-owned, is also enthusiastic about TZTC’s generosity for “contribut[ing] to the social well-being of many Zimbabweans” [46].

### 3.4. Institutions: Preferential Partnerships

As an overseas subsidiary of an SOE TZTC has close ties with the Chinese embassy in Harare and its Economic and Commercial Counsellor’s office, and regularly participates in events related to bilateral trade and cultural exchange [41]. Through such activities, it is able to build networks with policy-makers in both governments. CNTC has particularly cultivated a close relationship with the Zimbabwean Ministry of Agriculture through study exchanges, where personnel from the ministry and prominent tobacco farmers traveled to China’s major tobacco-growing province, Yunnan [59]. For example, in 2009, a consultation meeting of senior experts was held in Yunnan, convened by the local provincial tobacco companies, and attended by Zimbabwe Tobacco Research Board, the state regulatory body, and TZTC staff [60]. This was followed by numerous subsequent visits, and in 2014, experts from the two countries collaborated to grow Zimbabwe’s “golden leaf” in Yunnan.

TZTC’s ties and close relationships with Zimbabwe’s Ministry of Agriculture and other policy-makers have resulted in preferential treatment in Zimbabwe. For example, the government of Zimbabwe passed the Indigenisation and Economic Empowerment Act in 2010, by which all foreign-operated companies in Zimbabwe must relinquish 51% company shares to local Zimbabweans [61]. TZTC, even though fully Chinese-owned, was granted an exclusion from this law, which was attributed to the key role it played in Zimbabwe’s economy [40]. Facing public criticism for this decision, the former Indigenization Minister Saviour Kasukuwere stated, “Companies such as Tian Ze [TZTC] came into the country at a time when no one wanted to come in. They have been supporting our agriculture and our farmers, so we look at those things when considering whether to exempt them or not” [61].

TZTC has recently taken on the role of facilitating partnerships between other Chinese enterprises and the Zimbabwean government. In June 2019, at the request of the Zimbabwean government, CNTC agreed that TZTC would increase its tobacco exports from $500 US to $600 million US. However, the extra $100 million US would be earmarked to purchase heavy machinery and farming equipment from Zoomlion, one of China’s largest heavy machinery companies [62]. Zoomlion will provide the Zimbabwean government with equipment of equal value to the purchased tobacco leaf, with CNTC handling payments to Zoomlion, allowing Zimbabwe to purchase the necessary farming equipment without having to use precious foreign exchange reserves [62]. This scheme, dubbed “tobacco for equipment”, is described as designed to boost the Zimbabwean economy through improving production yields, increasing farm acreage, and improving farmer incomes. It has also further strengthened TZTC’s position in the tobacco industry as not only a leaf buyer but as a supplier of equipment essential for industry expansion.

## 4. Discussions

In the early 2000s, Zimbabwe’s tobacco industry was in tatters. While it is impossible to know if the ruin of the industry would have been permanent without investments from China, the role of CNTC in revitalizing Zimbabwe’s industry cannot be overstated. Previously operating companies had fled due to the violence targeting white farmers. Sanctions and political tensions limited their ability to rebuild and prevented other development partners from supporting rural development. China and CNTC stepped in when there were few other actors willing to help farmers in Zimbabwe. That tobacco-growing finds fertile ground in contexts of poverty and vulnerability is not a new finding, but an important one to remember for those who wish to reduce tobacco supply. Efforts need to not only include diversification for those currently growing tobacco but also prevent the growth of the industry by providing alternative options to farmers hoping to develop sustainable livelihoods.

Indeed, TZTC’s success in Zimbabwe can be largely attributed to its ability to capitalize on opportunities created by China—Zimbabwe development cooperation in a context of extreme economic need. While the Zimbabwean government saw TZTC as a development project able to rebuild the tobacco industry and provide employment to the rural populations, TZTC used the vast resources at its disposal as an overseas subsidiary of a powerful SOE to instigate a shift to contract farming in Zimbabwe of flue-cured Virginia leaf, leading to a sharp decline in the production of burley tobacco favored by other buyers. TZTC benefited from historically friendly bilateral relations between the governments of China and Zimbabwe, which aligned Zimbabwe’s Look-East policy with China’s position as an increasingly assertive player on the African continent. Consequently, CNTC, and by its extension TZTC, was uniquely positioned to engage in the tobacco sector in Zimbabwe, drawing on its political network to engage in policy-making both in China and Zimbabwe, and successfully embedding its interests at all levels of industrial development. These findings are significant for a number of reasons.

First, given that China continues to support tobacco farming as part of its overseas agricultural development work, it is imperative that the tobacco control community engage with development policy in both China and recipient states [10]. In this case, while TZTC has increased foreign exchange earnings for the Zimbabwean state, its impact on farmers’ livelihoods is questionable. Meanwhile, Chinese support for the industry has increased dependence on the tobacco industry for both farmers —who have become indebted to TZTC through contract farming arrangements—and the state who relies on China to buy its largest agricultural export. This dependence has negative consequences for both global health and agricultural development, the intersections of which tobacco control advocates rarely discuss, but might provide an additional opportunity to promote supply reduction policies. Emerging literature from other tobacco-growing countries of sub-Saharan Africa has called for an integration of tobacco control policies into the broader context of Sustainable Development Goals [63]. Such an approach might be further extended to communicate with exporting states and China about the wide-ranging, mostly negative, impacts of the industry on sustainable development and promote alternative industries for investment.

Second, the tobacco control community would benefit from a greater understanding of CNTC’s global supply chain and how its expansion strategy is impacting countries with CNTC operations. Despite being the largest tobacco company in the world, CNTC has remained under the radar of tobacco control researchers, quietly expanding since the early 2000s [2,64]. While numerous studies have been published on the globalization strategies of transnational tobacco companies, CNTC is unique due to the government backing it enjoys, and its ability to engage in policy-making both domestically and internationally. The case presented here is one of the first to explore how this unique advantage benefits CNTC’s overseas operations.

CNTC’s ability to capitalize on opportunities created by Chinese foreign policy is especially relevant given the adoption of the Belt and Road Initiative [65]. While the details of President Xi Jinping’s signature initiative remain opaque, it is aimed at increasing trade among other objectives, and development partners, such as the government of Zimbabwe, see it as an opportunity to deepen bilateral relations further [66]. CNTC has noted that some of the countries included are particularly attractive potential future markets [65]. The tobacco control community needs to monitor how CNTC might further expand through the initiative.

Lastly, these findings raise important questions for WHO FCTC implementation, as both China and Zimbabwe are signatories to the international treaty. Specifically, Article 5.3 addressing industry interference is especially relevant to China, as the government has yet to distance itself from the state-owned tobacco industry. By virtue of its structure as a powerful SOE, CNTC is the only tobacco industry in the world with the ability to directly engage in policy debates domestically and internationally when integrated as part of China’s development package. As this case shows, CNTC’s close relationship with state actors has implications outside of China as well as within, especially in countries engaged in development cooperation.

Both Zimbabwe and China also have an obligation to implement Article 17 to provide economic alternatives to tobacco farming [67]. As opposed to expanding the industry, the two countries might direct development resources to support diversification. In 2015, President Xi Jinping pledged US $60 billion to support agricultural modernization in sub-Saharan Africa, followed by the announcement of a further $60 billion US of development assistance in 2018 for eight key initiatives, including agricultural modernization [68]. Given China and Zimbabwe’s obligations as signatory countries to the FCTC, the negative impacts, and developmental implications of tobacco leaf farming, these resources would perhaps be best directed to agricultural industries other than tobacco.

## 5. Conclusions

China’s support for the tobacco industry as part of its agricultural cooperation with Zimbabwe is reflective of China’s unique approach to aid and development. As one of the only development partners willing to invest in Zimbabwe in the early 2000s, China emerged as the ideal partner to revive its tobacco industry, through the establishment of TZTC to procure flue-cured tobacco for the vast Chinese market. Favorable conditions, including the backing of the Chinese government, close bilateral ties, and alignment of political goals, have allowed TZTC to become one of the major stakeholders in the Zimbabwean tobacco industry. This has created a context where farmers are increasingly dependent on tobacco as a crop, and the government is dependent on China as an export destination.

The continued global expansion of CNTC continues to pose a threat to tobacco control efforts and therefore, global public health. This paper has sought to contribute to mitigating this threat by illuminating one component of CNTC’s supply-side expansion and critically assessing claims regarding tobacco farming as development. In order to promote public health, the global tobacco control community needs to strengthen engagement with development policies in order to counter tobacco industry expansion and support tobacco farmers in diversifying away from tobacco. While the majority of states and development actors do not engage with tobacco industry actors, one of the most prominent in sub-Saharan Africa—China—does. Tobacco control advocates need to engage with Chinese foreign policy in Africa to discourage this support and suggest it be redirected to more health-promoting sectors, such as improving food security. Efforts could also focus on providing alternative livelihoods to not only current tobacco farmers but also those at risk of becoming tobacco farmers due to poverty. Such efforts would advance tobacco supply reduction, public health, and sustainable development goals.

## Figures and Tables

**Table 1:** Coding Framework.

Material Capabilities	Ideational Influences	Institutional Influences
- Historic bilateral trade & aid - Financial Investments - Creation of TZTC	- Reputation as a friend & partner - Corporate Social Responsibility initiatives	- State networks - Study exchanges - Facilitating partnerships
